# Design and Fabrication of Tryptophan Sensor Using Voltammetric Method

**DOI:** 10.3390/mi15081047

**Published:** 2024-08-18

**Authors:** Mohd Quasim Khan, Khursheed Ahmad, Rais Ahmad Khan

**Affiliations:** 1 Department of Chemistry, M.M.D.C, Moradabad, M.J.P. Rohilkhand University, Bareilly 244001, U.P., India; 2 School of Materials Science and Engineering, Yeungnam University, Gyeongsan 38541, Republic of Korea; 3 Department of Chemistry, College of Science, King Saud University, Riyadh 11451, Saudi Arabia

**Keywords:** Ni-WO_3_, tryptophan, differential pulse voltammetry, sensor

## Abstract

L-tryptophan is an amino acid that significantly impacts metabolic activity in both humans and herbivorous animals. It is also known as a precursor for melatonin and serotonin, and its levels must be regulated in the human body. Therefore, there is a need to develop a cost-effective, simple, sensitive, and selective method for detecting L-tryptophan. Herein, we report the fabrication of an L-tryptophan sensor using a nickel-doped tungsten oxide ceramic-modified electrode. The Ni-WO_3_ was synthesized using simple strategies and characterized by various advanced techniques such as powder X-ray diffraction, scanning electron microscopy, energy-dispersive X-ray spectroscopy, and photoelectron X-ray spectroscopy. Furthermore, a glassy carbon electrode was modified with the synthesized Ni-WO_3_ and explored as the L-tryptophan (L-TRP) sensor. Cyclic voltammetry and differential pulse voltammetry were used to investigate the sensing ability of the modified electrode (Ni-WO_3_/GC). The Ni-WO_3_/GC exhibited an excellent limit of detection of 0.4 µM with a good dynamic linear range. The Ni-WO_3_/GC also demonstrated excellent selectivity in the presence of various electroactive molecules. The Ni-WO_3_/GC also showed decent reproducibility, repeatability, stability, and storage stability. This work proposes the fabrication of novel Ni-WO_3_/GC for the sensing of L-tryptophan. So far, no report is available on the use of Ni-WO_3_/GC for the sensing of L-TRP. This is the first report on the use of Ni-WO_3_/GC for the sensing of L-TRP sensing applications.

## 1. Introduction

L-tryptophan (L-TRP) is an essential amino acid that plays a pivotal role in protein synthesis and serves as a precursor for the biosynthesis of serotonin and melatonin, critical neurotransmitters involved in regulating mood, sleep, and other physiological functions [1,2,3,4]. As humans cannot synthesize L-TRP endogenously, it must be obtained through diet, with the World Health Organization (WHO) recommending a daily intake of approximately 4 mg per kilogram of body weight [5]. Proper L-TRP metabolism is vital for maintaining health, and imbalances can lead to serious conditions such as schizophrenia, Alzheimer’s disease, Parkinson’s disease, and depression [6]. Given the importance of L-TRP in various biological processes and its link to multiple diseases, accurate and efficient detection methods are crucial for clinical diagnostics and health monitoring [7,8]. Previously, conventional methods such as high-performance liquid chromatography (HPLC), chemiluminescence, spectrofluorometry, and capillary electrophoresis were widely used for the determination of L-TRP. HPLC is widely used due to its high accuracy and sensitivity, but often requires complex sample preparation and expensive equipment [9,10,11,12]. Chemiluminescence methods offer high sensitivity and specificity but can be affected by quenching agents present in the sample matrix [13]. Spectrofluorometry is another common technique, valued for its simplicity and rapid analysis, although it can suffer from interference from other fluorescent species [14]. Thus, alternative techniques and methods are required to overcome the issue of conventional sensing techniques. In this regard, an electrochemical method has been developed for the efficient detection of L-TRP with good selectivity. Electrochemical sensors have emerged as a promising approach due to their potential for rapid, on-site analysis, eco-friendliness, and decent repeatability, with high sensitivity and selectivity [15,16,17,18,19,20]. Electrochemical sensors operate based on the redox reactions of analytes at the electrode surface, providing chemical information in an electrical signal [21,22,23,24]. These sensors have received extensive attention because of their low cost, portability, and ease of miniaturization, making them suitable for point-of-care applications [25]. Recent advancements have focused on enhancing the performance of these sensors through the development of novel electrode materials and surface modifications to improve sensitivity, selectivity, and stability [26]. Incorporating nanomaterials and catalysts into the electrode design has shown significant promise in achieving these goals. Electrode materials (catalysts) play a critical role in electrochemical sensors by lowering the activation energy of redox reactions, thereby increasing the sensitivity and response time of the sensor [23].

Previously, Naganathan et al. [2] described an easy synthesis method for Ce-doped ZnO/f-MWCNT, which was used to construct the L-TRP sensor. This sensor exhibited a decent sensitivity of 2.59 μA/nM.cm^2^ [2]. Similarly, Zeinali et al. [14] proposed the L-TRP sensor using a ternary composite (SnO_2_-Co_3_O_4_@rGO) as the electrode material, which also demonstrated decent performance [14]. The improved performance of the proposed material was attributed to the conductive properties of rGO and the synergistic interactions within the ternary composite [14]. Peter and his research team have developed an L-TRP sensor using CuO nanoflakes anchored on a polythiophene nanocomposite as the electrode modifier [27]. The sulfur groups of polythiophene interact with the carboxylic acid and amine groups of L-TRP, enhancing the conductance of the modified electrode. In another report, Nie et al. [15] investigated the electrochemical sensing performance of CeO_2_/ERGO/GCE for the sensing of L-TRP, which exhibited decent electrochemical performance for the detection of L-TRP. Gao et al. [28] synthesized a ternary composite of polydopamine/graphene/MnO_2_ for L-TRP detection and reported a decent limit of detection (LOD) of 0.39 μM. Haldorai et al. [17] also explored the sensing properties of the rGO/SnO_2_ composite-modified electrode for the monitoring of L-TRP. Yang et al. [16] constructed nitrogen-doped carbon@TiO_2_ double-shelled hollow spheres for TRP determination, while Murugan et al. [15] employed a SnS/TiO_2_@GO composite coated GCE as an L-TRP sensor. Sun et al. [18] developed the L-TRP sensor using a NiO/CNT/PEDOT composite as the electrode material, while Fan et al. [29] utilized a Nafion/TiO_2_-graphene composite film for L-TRP sensing applications. Yola et al. [3] also reported a novel L-TRP sensor employing a polyoxometalate/rGO-modified electrode. Another study proposed the functionalized graphene quantum dots/Bi-metallic composite modified electrode as an L-TRP sensor [13]. Each catalyst material offers unique advantages but also presents specific challenges, such as cost, stability, and selectivity issues. The continuous search for cost-effective, highly efficient, and stable catalysts drives ongoing research in this field.

Tungsten trioxide (WO_3_) is a well-known semiconductor material with excellent electrochemical properties, making it a popular choice for sensor applications [30,31,32,33,34]. Doping WO_3_ with nickel (Ni) may be a promising approach for the enhancement of its catalytic activity and electrical conductivity for electrochemical sensing applications. The use of Ni-WO_3_ as a catalyst in electrochemical sensors for L-TRP detection is a novel approach aimed at leveraging the enhanced properties of the doped material. The hypothesis is that Ni doping may improve the sensitivity, selectivity, and stability of WO_3_-based sensors by providing more active sites for L-TRP oxidation and facilitating faster electron transfer.

In this study, we reported the synthesis of Ni-WO_3_, which is characterized by various sophisticated physicochemical properties. The synthesized Ni-WO_3_ was further used as a catalyst for the construction of the L-TRP sensor. The Ni-WO_3_-modified electrode exhibits decent performance and sensitivity. According to the best of our literature survey, no report was found on the use of Ni-WO_3_ as a catalyst for the construction of the L-TRP sensor. This is the first report that proposed the fabrication of an L-TRP sensor using Ni-WO_3_ as a catalyst.

## 2. Experimental Section

### 2.1. Chemicals

Sodium tungstate dehydrate (Na_2_WO_4_·2H_2_O; ACS reagent, ≥99%), L-tryptophan (L-TRP), nickel nitrate hexahydrate (Ni(NO_3_)_2_·6H_2_O; 99.999% trace metals basis), and hydrogen peroxide (H_2_O_2_; 30 wt. % in H_2_O, ACS reagent) were bought from Merck, India. All the other chemicals were purchased from Sigma and Alfa Aesar, India. Phosphate-buffered saline (PBS) solutions were purchased from Sigma, India. All the chemicals and reagents were of analytical quality and have been used without any further purification.

### 2.2. Synthesis of Ni-WO_3_

**Scheme 1:**
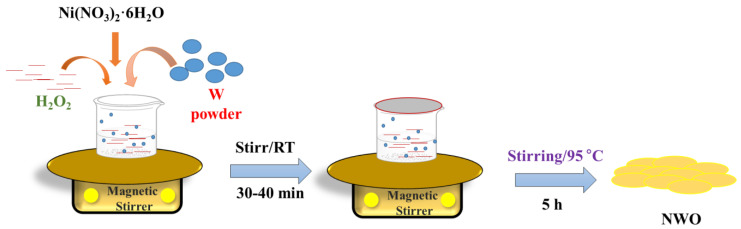
Schematic description for the preparation of WO and NWO.

WO and NWO were synthesized by employing the sol–gel method. For the NWO sample, typically, 6.5 g of tungsten (W) powder was slowly dissolved in 90 mL of hydrogen peroxide (H_2_O_2_; 30% in H_2_O) in a beaker as shown in Scheme 1. A total of 0.45 g of nickel nitrate hexahydrate (Ni(NO_3_)·6H_2_O) was slowly added to this reaction solution and stirred for 30–40 min at room temperature (RT). After the complete dissolution of the precursors, the temperature of the magnetic stirrer was set to 95 °C and stirred for another 5 h. A pale yellow solution was observed.

Further, the solvent was evaporated, and a yellow-colored powder was obtained, which was washed with deionized (DI) water and ethanol via the centrifuge method. The WO sample was also prepared in similar conditions, except for the addition of Ni-source. The obtained products were dried at 60–70 °C for 6–8 h.

### 2.3. Instrumental

The X-ray diffraction (XRD) graphs of the synthesized WO and NWO samples were obtained using Rigaku, RINT, and JAPAN (2500 V) X-ray diffractometers (irradiation = Cu Kα, λ = 1.5406 Å). The scanning electron microscopic (SEM) images of the synthesized WO and NWO samples have been recorded using the Zeiss Field Emission Scanning Electron Microscopy (FE-SEM) Supra 55 instrument. The energy dispersive X-ray (EDX) results of the WO and NWO samples were obtained using Oxford Instrument’s X-max Aztec EDX instrument connected with the FE-SEM instrument. The X-ray photoelectron spectroscopy (XPS) analysis was carried out using the Thermo Fisher Scientific XPS instrument. The electrochemical analysis was carried out using the CH instrument, which involves three electrode assemblies. The WO or NWO-modified glassy carbon (GC) electrodes were used as working electrodes, Ag/AgCl as reference electrodes, and Pt wire-based electrodes as counter electrodes.

### 2.4. Electrode Modification

**Scheme 2:**
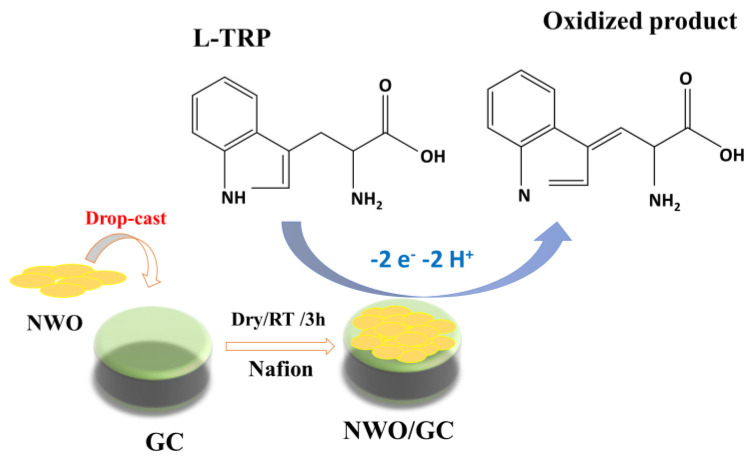
Schematic illustration for the fabrication of NWO/GC and its working for L-TRP sensing.

The WO or NWO ink was prepared by dispersing 4 mg catalyst (WO or NWO) in 3 mL of DI water and 0.1% Nafion, which was sonicated for 1 h at RT. The obtained ink was coated (6 µL) on the GC electrode by the drop-caste method and dried at RT for 3 h (Scheme 2). The WO or NWO-modified GC electrodes have been denoted as WO/GC and NWO/GC, respectively.

## 3. Results and Discussion

### 3.1. Materials Characterization

**Figure 1:**
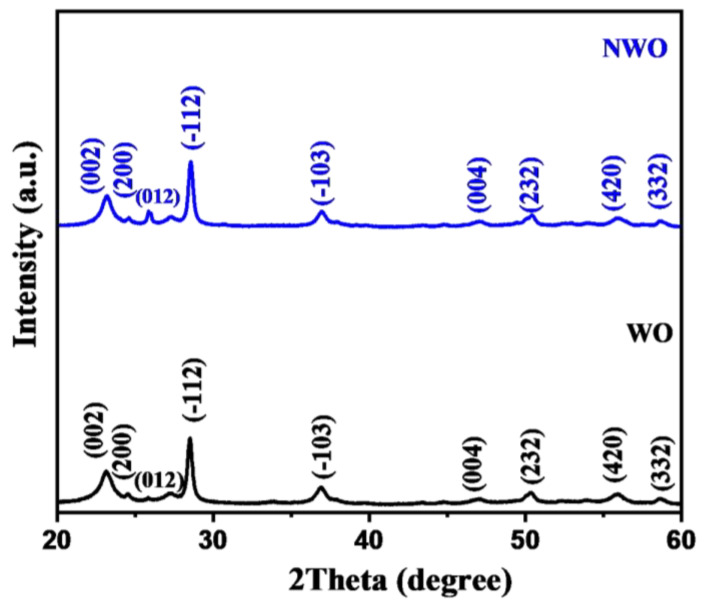
XRD patterns of the synthesized WO and NWO.

XRD is one of the most efficient techniques to study the crystalline, amorphous nature, and phase of transition metal oxides. Thus, we adopted the XRD method to know the phase purity, crystalline nature, and formation of the prepared WO and NWO samples. Figure 1 depicts the recorded XRD graphs of the WO and NWO samples. It is seen that the WO sample exhibits various diffraction peaks, which can be indexed to the well-known (002), (200), (012), (−112), (−103), (004), (232), (420), and (332) diffraction planes, respectively.

The XRD pattern confirmed the formation of hexagonal WO_3_ with a good crystalline nature. The XRD pattern of the NWO sample also demonstrated the similar diffraction planes of (002), (200), (012), (−112), (−103), (004), (232), (420), and (332). This obtained XRD pattern was in good agreement with the reported JCPDS card data number 83-0950. This indicates that WO and NWO are successfully formed with reasonably good phase purity, which was indicated by the absence of any other diffraction peak related to the residual or impurity materials. The average crystallite size of the WO and NWO samples was determined by employing the Scherer equation, which is provided below in Equation (1),

(1) $$D=\frac{0.9\;\lambda }{\beta \;\cos\theta }$$

In Equation (1), D is the crystallite size (nm) of prepared WO and NWO, λ is the wavelength (1.54 Å), θ = diffraction angle, and β is the half-width of the XRD peaks.

The crystallite size of the synthesized WO was found to be 8.6 nm, and NWO shows a crystallite size of 10.6 nm.

**Figure 2:**
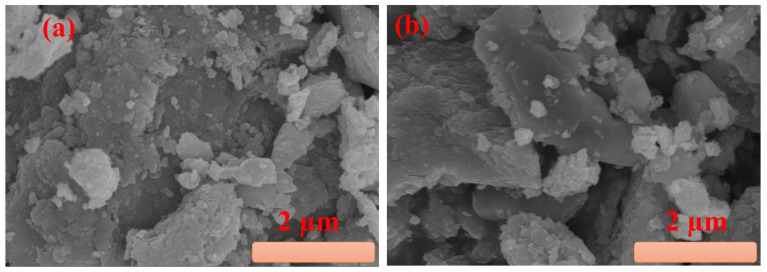
FE-SEM images of the synthesized WO (**a**) and NWO (**b**).

The morphology of the catalysts has a significant role in the electron transport process. Thus, the morphology of the prepared WO and NWO needs to be investigated. In this context, surface structural images of the synthesized WO and NWO were studied by employing SEM analysis. The SEM micrograph of the WO is shown in Figure 2a, which reveals that the WO is composed of small nanoparticles that are agglomerated. The SEM picture of the NWO has been depicted in Figure 2b, and similar surface morphological features have been observed. It is expected that the synthesized WO or NWO may be beneficial for the catalysis and electrocatalysis processes. The above SEM analysis clearly indicated the particle surface morphological properties of the WO and NWO samples.

**Figure 3:**
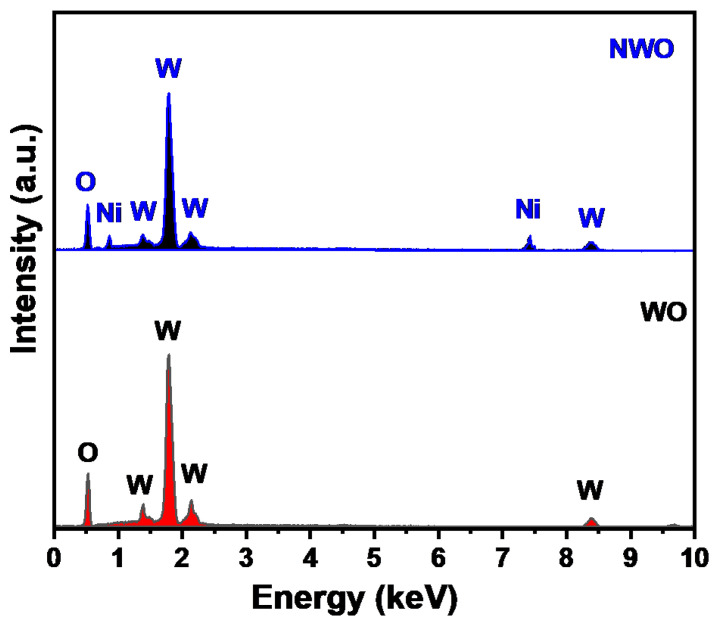
EDX spectra of the prepared WO and NWO samples.

However, the presence of elements such as Ni, W, and O still needs to be confirmed. In this regard, another technique needs to be employed, and in this regard, we adopted the EDX approach for the elemental determination in the WO and NWO samples. The EDX spectrum of the WO is shown in Figure 3, which indicates the signals for the W and O elements. The EDX spectrum of the NWO sample is depicted in Figure 3, and three signals appeared, which correspond to the presence of Ni, W, and O elements. The EDX investigations authenticated the presence of Ni in the prepared NWO sample.

**Figure 4:**
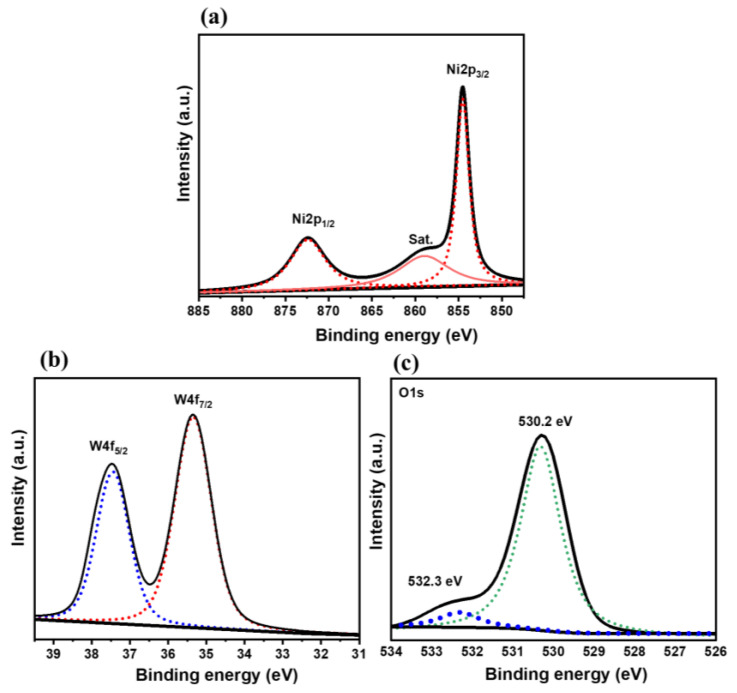
XPS study: High-resolution Ni2p (**a**), W4f (**b**), and O1s (**c**) XPS spectra of the synthesized NWO.

The XPS technique was also utilized to further understand the elemental oxidation states in the prepared NWO sample. The high-resolution XPS scan of the Ni2p has been demonstrated in Figure 4a. The XPS scan exhibits the presence of two major XPS signals at the binding energy values of 854.4 and 872.8 eV, which can be attributed to the presence of Ni2p_3/2_ and Ni2p_1/2_, respectively.

The XPS scan for W4f shows the presence of two XPS peaks at binding energies of 37.46 and 35.36 eV, which are ascribed to the presence of W4f_5/2_ and W4f_7/2_, respectively. Figure 4c presents the XPS spectrum of the O1s element. The O1s exhibit two deconvoluted peaks at binding energy values of 532.3 and 530.2 eV, which can be attributed to the presence of hydroxyl groups and lattice oxygen, respectively. The obtained XPS results are in good agreement with the previously reported literature [35].

### 3.2. ElectroCatalytic Properties of WO/GC and NWO/GC

**Figure 5:**
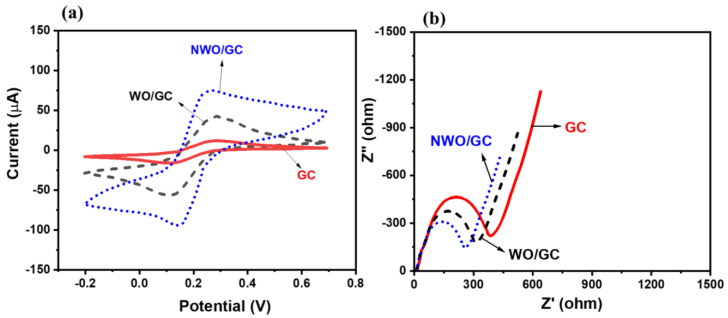
CVs (**a**) and Nyquist plots (**b**) of the bare GC, WO/GC, and NWO/GC electrodes (conditions: 5 mM [Fe(CN_6_)]^3−/4−^ redox system; 0.1 M KCl; scan rate = 50 mV/s).

The catalytic properties of the modified WO/GC and NWO/GC electrodes have been studied by employing CV technology. The CV of the WO/GC and NWO/GC have been recorded in the presence of a 5 mM [Fe(CN_6_)]^3-/4-^ redox system (0.1 M KCl; scan rate = 50 mV/s). The obtained CV graphs of the WO/GC and NWO/GC are shown in Figure 5a. It can be observed that WO/GC has a low current response for the redox peaks in comparison to the NWO/GC electrode. The incorporation of Ni may provide additional charge carriers and facilitate electron mobility. Thus, it is also clear that Ni-doping may enhance the electrical conductivity of the pristine WO_3_. Thus, it is understood that NWO/GC electrodes possess higher electrocatalytic properties, which may arise due to the presence of synergistic interactions between Ni-W-O elements and the improved electrical conductivity of the NWO/GC electrode. The CV pattern of the bare GC electrode was also recorded under similar conditions, and it has been observed that the bare GC electrode has a lower current response compared to the NWO/GC electrode. This indicated that NWO has decent electrocatalytic properties and improved the current response for electrochemical reactions. The electrochemical impedance spectroscopic (EIS) investigations were also carried out using a 5 mM [Fe(CN_6_)]^3−/4−^ redox system (0.1 M KCl; scan rate = 50 mV/s). The Nyquist curves were recorded for the bare GC, WO/GC, and NWO/GC electrodes in the above-mentioned redox system. The bare GC exhibits a higher charge transfer resistance (R_ct_) value of 390.1 Ω, whereas the WO/GC electrode shows a relatively lower R_ct_ value of 314.2 Ω. This indicates that WO/GC has higher electrical conductivity compared to the bare GC (Figure 5b). The NWO/GC electrode exhibited the lowest R_ct_ value of 260.3 Ω compared to the bare GC or WO/GC electrode. This confirmed that the NWO/GC electrode has a higher electrical conductivity and is a promising electrode for sensing applications.

### 3.3. Electrochemical Sensing Performance of WO/GC and NWO/GC

**Figure 6:**
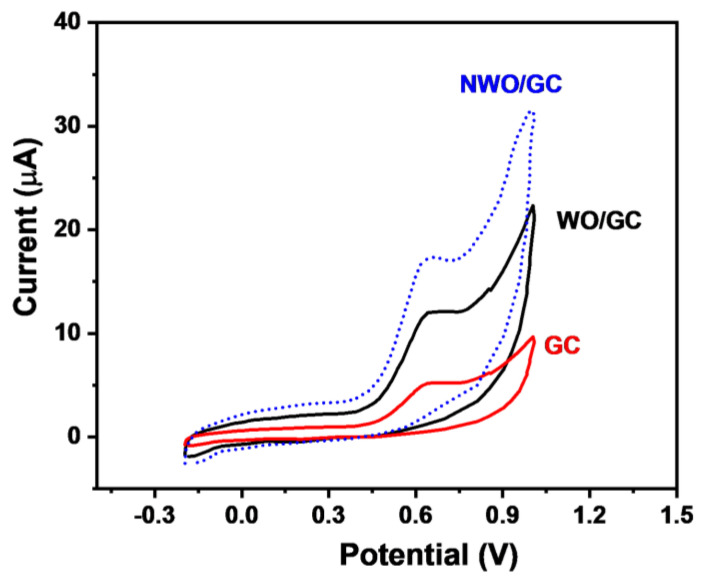
CVs of the WO/GC and NWO/GC electrodes in 200 µM L-TRP in 0.1 M PBS (pH = 7.0; scan rate = 50 mV/s).

The EIS and CV results clearly showed that the NWO/GC electrode has superior activity compared to the bare GC or WO/GC electrode. The NWO/GC may be used as an efficient working electrode for the sensing of L-TRP using three electrode assemblies. Thus, we have studied the sensing performance of the NWO/GC electrode as an L-TRP sensor. Moreover, the performance of the bare GC and WO/GC electrodes was also compared with that of the NWO/GC electrode. The CV of the bare GC, WO/GC, and NWO/GC electrodes was collected using three electrode assemblies for 200 µM L-TRP in 0.1 M PBS (pH = 7.0; scan rate = 50 mV/s). The pH of the 0.1 M PBS was also varied to obtain a higher current response for the determination of L-TRP. Figure S1 shows that the NWO/GC electrode has a higher current response for the detection of L-TRP in 0.1 M PBS of pH 7.0. Figure 6 shows the obtained CV patterns of the bare GC, WO/GC, and NWO/GC electrodes, which revealed that the performance of the bare GC can be neglected due to poor current response (5.4 µA), whereas the WO/GC electrode exhibits considerable current response (12.07 µA) for the oxidation of L-TRP. In further investigations, the NWO/GC electrode demonstrated the highest current value (17.47 µA) for the oxidation of L-TRP, suggesting its excellent ability for the efficient determination of L-TRP, which may be ascribed to the higher electrical conductivity and synergistic interactions in the NWO/GC electrode. The NWO/GC electrode exhibited approximately 3.2 times higher current response compared to the bare GC electrode. Thus, it is considered that the NWO/GC electrode is the most efficient working electrode for the sensing of L-TRP compared to the bare GC or WO/GC electrode, and we selected the NWO/GC electrode as the working electrode for further electrochemical investigations such as the influence of scan rates, repeatability, storage stability, and reproducibility by employing the CV method.

**Figure 7:**
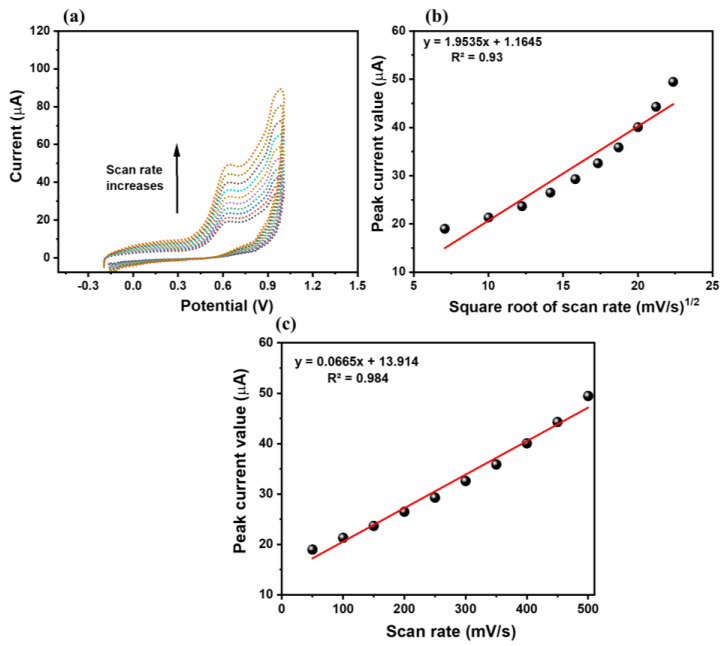
(**a**) CVs of the NWO/GC electrode in 200 µM L-TRP in 0.1 M PBS (pH = 7.0) at different scan rates (50–500 mV/s). (**b**) Corresponding calibration curves peak current versus square root of applied scan rate (**b**) and applied scan rate (**c**).

The CV patterns of the NWO/GC electrode have been recorded in 200 µM L-TRP in 0.1 M PBS (pH = 7.0) at different scan rates (50–500 mV/s). The obtained CV patterns of the NWO/GC electrode for 200 µM L-TRP at different applied scan rates are shown in Figure 7a. It can be observed from the obtained CV patterns that the current response for the oxidation of L-TRP increases with an increasing scan rate of 50–500 mV/s. Thus, it is clear that the current response directly increases with respect to the applied scan rates. The calibration curve between the oxidation peak current and the square root of applied scan rates is calibrated and has been presented in Figure 7b. A regression coefficient (R^2^) value of 0.92 was observed. The calibration curve between current responses and the applied scan rate is shown in Figure 7c. The R^2^ value of 0.99 has been observed, which suggests that the sensing of L-TRP is an adsorption-controlled process.

**Figure 8:**
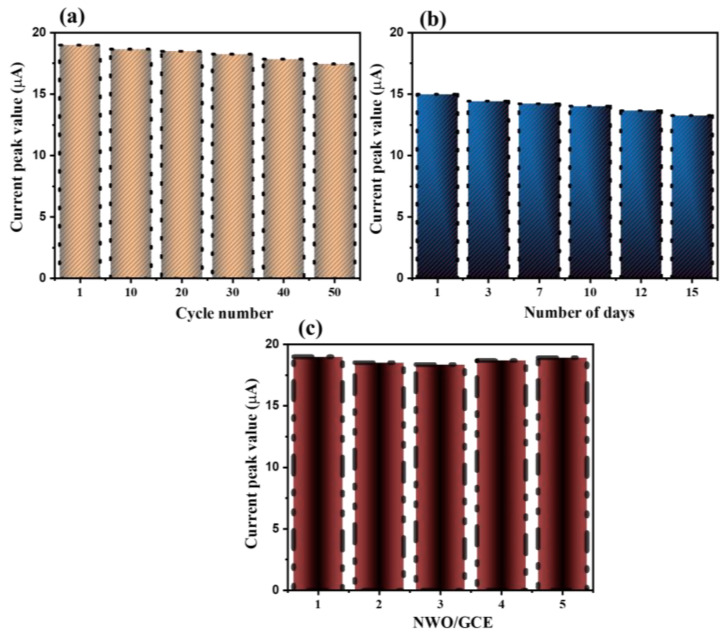
Repeatability (**a**), storage stability (**b**), and reproducibility (**c**) of the NWO/GC electrode in 200 µM L-TRP in 0.1 M PBS (pH = 7.0; scan rate = 50 mV/s).

Repeatability is one of the desirable characteristics of electrochemical sensors that needs to be studied. In this context, a repeatability study has been conducted by employing the CV method for the determination of 200 µM L-TRP in 0.1 M PBS (pH = 7.0; scan rate = 50 mV/s) using the NWO/GC electrode. Figure 8a shows the repeatability results of the NWO/GC electrode, which showed decent repeatability with an acceptable relative standard deviation (RSD) value of 3.76%.

Furthermore, the storage stability of the NWO/GC electrode was also studied under similar conditions used for repeatability, and it can be seen in Figure 8b that the NWO/GC electrode has a good storage stability of 15 days with insignificant degradation in performance after 15 days. For reproducibility purposes, five different GC electrodes were modified using NWO under similar conditions, and their performance was evaluated for 200 µM L-TRP in 0.1 M PBS (pH = 7.0; scan rate = 50 mV/s). The obtained results are compiled in Figure 8c, which exhibited negligible changes in the current responses, which indicated decent reproducibility. The probable mechanism for the electro-oxidation of L-TRP has been illustrated in Scheme 2. It can be seen that the electro-oxidation process of L-TRP involves the release of two electrons and two protons. In previous reports, it has been proposed that differential pulse voltammetry (DPV) is one of the most sensitive techniques compared to CV and is widely used for the determination of various biomolecules or hazardous materials. Thus, we have further used the DPV technique for the determination of L-TRP. The DPV curves of the NWO/GC electrode are recorded in the presence of different concentrations of L-TRP (0.5–200 µM) at a scan rate of 50 mV/s.

**Figure 9:**
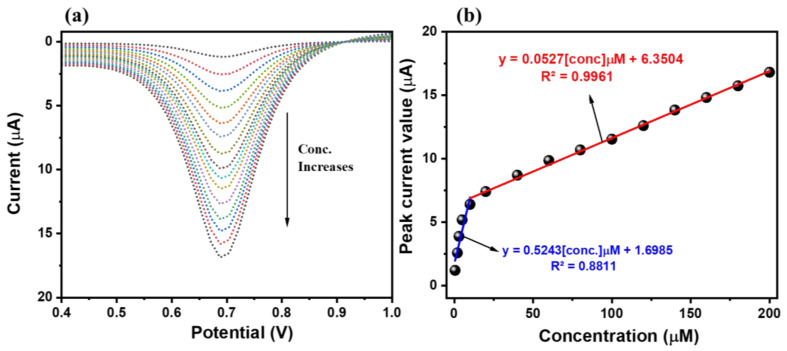
(**a**) DPV curves of the NWO/GC electrode in different concentrations (0.5, 2, 3, 5, 10, 20, 40, 60, 80, 100, 120, 140, 160, 180, and 200 µM) of the L-TRP (0.5–200 µM) in 0.1 M PBS (pH = 7.0; scan rate = 50 mV/s). (**b**) Calibration curve of the NWO/GC electrode between current responses and concentrations of L-TRP.

The obtained DPV patterns of the NWO/GC electrode are shown in Figure 9a. It is observed that current responses increase with increasing concentrations of L-TRP from 0.5 µM to 200 µM under the fixed applied scan rate of 50 mV/s. The calibration plot has been drawn between the peak current responses and the concentration of L-TRP, as depicted in Figure 9b. The current responses linearly increase with respect to the concentration of L-TRP, which is confirmed by an R^2^ value of 0.99. The limit of detection, which can be labeled as the LOD of the NWO/GC electrode, was determined by employing the following formula, given below:

LOD = 3.3 × σ_e_/slope (2)

The sensitivity of the NWO/GC electrode was calculated by employing Equation (3) given below

Sensitivity = S_e_/Area (3)

where σ_e_ is the standard error value, whereas S_e_ is the slope.

The LOD and sensitivity of the NWO/GC electrode for the determination of L-TRP were found to be 0.4 µM and 3.74 µA/µMcm^2^, respectively. The LOD and sensitivity of the NWO/GC electrode are compared with previous reports in Table 1, which indicated that the NWO/GC electrode has decent performance compared to the previous studies.

**Figure 10:**
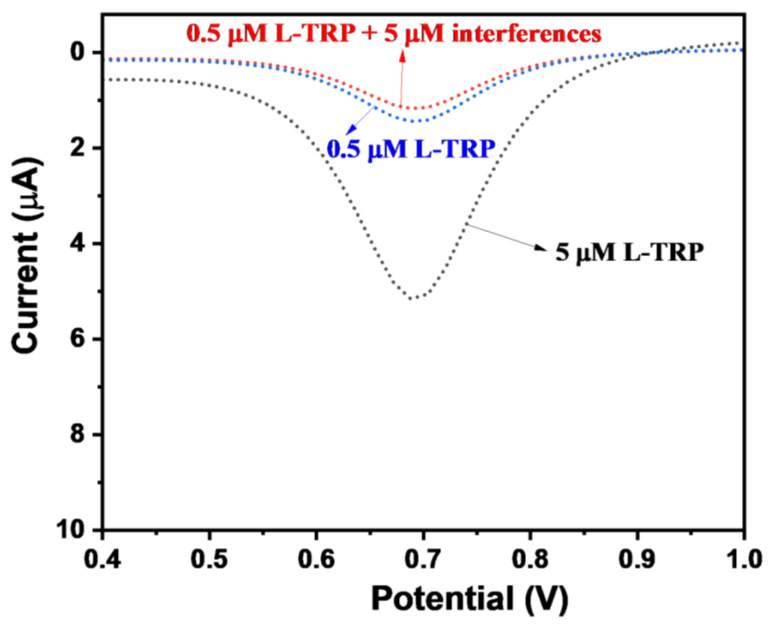
DPV curves of the NWO/GC electrode in 0.5 µM L-TRP, 5 µM L-TRP, and 0.5 µM L-TRP + 5 µM interfering molecules (chlorophenol, ascorbic acid, glucose, urea, dopamine, hydrazine, Ca^2+^, Mg^2+^, Cl^−^, and hydrogen peroxide) in 0.1 M PBS (pH = 7.0; scan rate = 50 mV/s).

Selectivity is also an important challenge for electrochemical sensors, and we have studied the selectivity of the NWO/GC electrode for the determination of L-TRP in the presence of various electroactive molecules. The DPV response of the NWO/GC electrode has been recorded for 0.5 µM L-TRP at an applied scan rate of 50 mV/s. Furthermore, the DPV pattern of the 0.5 µM L-TRP + 5 µM interfering molecules (chlorophenol, ascorbic acid, glucose, urea, dopamine, hydrazine, Ca^2+^, Mg^2+^, Cl^−^, and hydrogen peroxide) was obtained at an applied scan rate of 50 mV/s (Figure 10).

The observations showed that very little change could be observed in the DPV response, which can be neglected. This indicates that the NWO/GC electrode has a decent selective nature for the efficient determination of L-TRP in the presence of various interfering molecules (chlorophenol, ascorbic acid, glucose, urea, dopamine, hydrazine, Ca^2+^, Mg^2+^, Cl^−^, and hydrogen peroxide). The selectivity test was also conducted at higher concentrations of L-TRP and interfering species. The DPV of the NWO/GC was also obtained in the presence of 0.2 mM L-TRP + 1 mM AA + DA + UA, as shown in Figure S2. It is seen that a higher concentration of the interfering materials could not significantly affect the performance of the NWO/GC electrode. The presence of tyrosine (Tyr) + methionine (Met) + cysteine (Cys) in the L-TRP solution did not influence the performance of the NWO/GC electrode, as shown in Figure S2. Thus, we can summarize that the NWO/GC electrode is a promising candidate for the selective determination of L-TRP. The real sample investigations were carried out using a urine sample via the standard addition method. The urine sample of the 24-year-old healthy person was collected with his permission. The DPV of the NWO/GC electrode was checked in the urine sample. Furthermore, 0.1 mM L-TRP was spiked in the urine sample, and its DPV response was checked (Figure S3). The obtained results showed that NWO/GC has recoveries of 97.5%, which is acceptable.

## 4. Conclusions

In this section, it is necessary to compile that NWO and WO are synthesized by employing a simple synthesis procedure and characterized by various sophisticated techniques. The synthesized NWO was further applied as a catalyst for the construction of the L-TRP sensor. The active area of the GC electrode was coated via the drop-caste method, and electrochemical sensing performance was studied by employing CV and DPV methods. The CV results demonstrated that the NWO/GC electrode has higher electrochemical activity for the sensing of L-TRP than the bare GC or WO/GC electrodes. Furthermore, DPV results suggested that NWO/GC is a promising candidate for the efficient and selective determination of L-TRP. The NWO/GC also exhibited good storage stability and repeatability for the sensing of L-TRP. It is believed that NWO/GC may be an efficient working electrode for the construction of an L-TRP sensor. The real sample study also exhibited good catalytic properties in the fabricated NWO/GC electrode. Finally, we state that the present study proposes a simple and eco-friendly synthesis for TRP sensors. We believe that NWO/GC may be used for the construction of an L-TRP sensor, or its performance may be further improved by introducing novel metal carbide or nitride, such as titanium carbide, MXene, etc., in the proposed working electrode.

## Figures and Tables

**Table 1:** Comparison of the electrochemical sensing performance of the NWO/GC electrode for the sensing of L-TRP with previous studies [7,18,36,37,38].

Catalyst	LOD (µM)	Linear Range (µM)	References
NWO/GC	0.1	0.5–10, 10–200	This study
NiO/CNT/PEDOT/GCE	0.21	1–41	18
β-CD/CQDs/GCE	0.16	5–270	7
Ni-ZIF-8/N S-CNTs/CS/GCE	0.69	5–850	36
QDs/SiO_2_-MIP	0.35	0–8	37
Au-MWCNTs	3	-	38

## Data Availability

Authors elect to not to share data.

## Supplementary Materials

The following supporting information can be downloaded at: https://www.mdpi.com/article/10.3390/mi15081047/s1, Figure S1. Current values of the NWO/GC electrode in 200 µM L-TRP in 0.1 M PBS of different pH (pH = 3, 5, 7 and 9) at scan rates (50 mV/s). Figure S2. DPV curve of the NWO/GC in 0.2 mM L-TRP, 0.2 mM L-TRP + 1 mM AA+DA+UA, 0.2 mM L-TRP + 1 mM Tyr + Met, and 0.2 mM L-TRP + 1 mM Tyr + Met + Cys in 0.1 M PBS (pH 7.0; scan rate = 50 mV/s). Figure S3. DPV curve of the NWO/GC in 0.1 mM L-TRP in urine sample (scan rate = 50 mV/s). Inset shows urine sample.
