# Supplementary material for: Design and Fabrication of Tryptophan Sensor Using Voltammetric Method
Source: Micromachines (Basel). 2024 Aug 18;15(8):1047. doi: 10.3390/mi15081047 (PMC11356631; doi:10.3390/mi15081047)
Supplement: Supplementary file 1 [file micromachines-15-01047-s001.zip › micromachines-3114533-supplementary.pdf]

micromachines

Supplementary Materials

Design and Fabrication of Tryptophan Sensor Using Voltammetric Method

Mohd Quasim Khan [1], Khursheed Ahmad [2,*] and Rais Ahmad Khan [3]

1 Department of Chemistry, M.M.D.C, Moradabad, M.J.P. Rohilkhand University, Bareilly 244001, U.P., India; quasimkhanmohd90@gmail.com
2 School of Materials Science and Engineering, Yeungnam University, Gyeongsan 38541, Republic of Korea
3 Department of Chemistry, College of Science, King Saud University, Riyadh 11451, Saudi Arabia; krais@ksu.edu.sa
* Correspondence: khursheed@yu.ac.kr

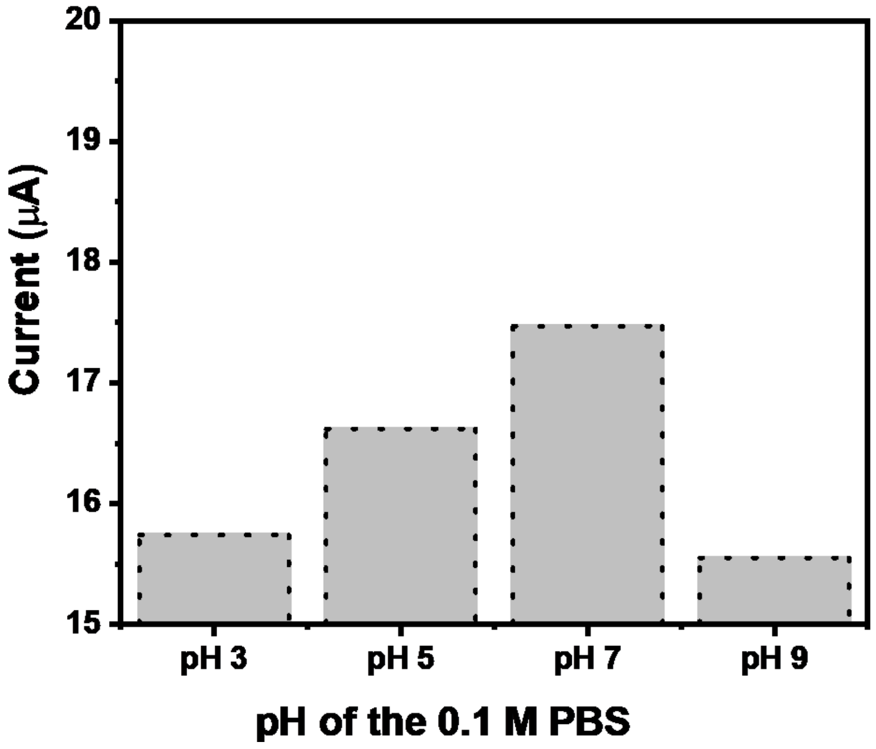

Figure S1. Current values of the NWO/GC electrode in 200 μM L-TRP in 0.1 M PBS of different pH (pH = 3, 5, 7 and 9) at scan rates (50 mV/s).

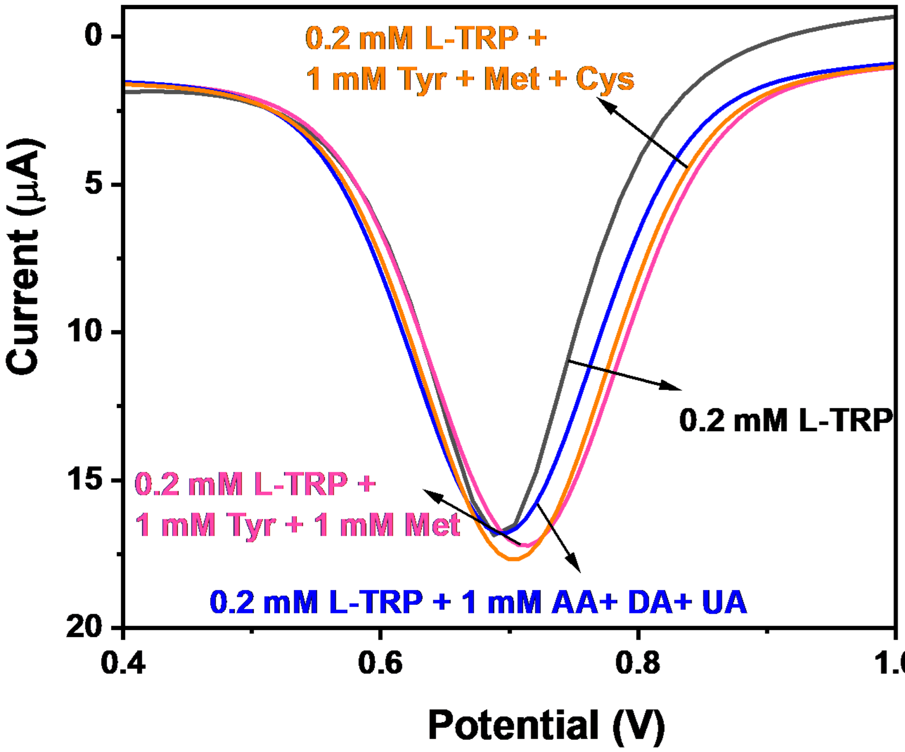

Figure S2. DPV curve of the NWO/GC in 0.2 mM L-TRP, 0.2 mM L-TRP + 1 mM AA+DA+UA, 0.2 mM L-TRP + 1 mM Tyr+Met, and 0.2 mM L-TRP + 1 mM Tyr+Met+Cys in 0.1 M PBS (pH 7.0; scan rate = 50 mV/s).

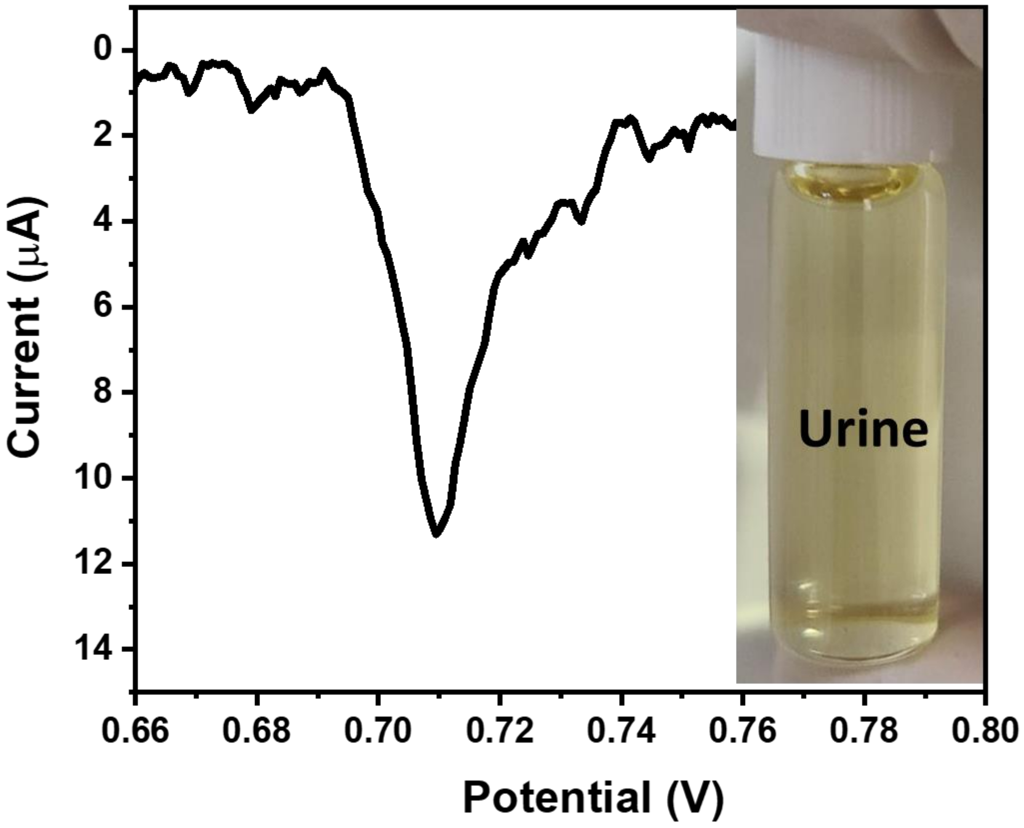

Figure S3. DPV curve of the NWO/GC in 0.1 mM L-TRP in urine sample (scan rate = 50 mV/s). Inset shows urine sample.
